# Intimate Partner Violence: A Risk Factor for Gestational Diabetes

**DOI:** 10.3390/ijerph17217843

**Published:** 2020-10-26

**Authors:** Carmen Pheiffer, Stephanie Dias, Sumaiya Adam

**Affiliations:** 1 Biomedical Research and Innovation Platform (BRIP), South African Medical Research Council, P.O. Box 19070, Tygerberg, Cape Town 7505, South Africa; stephanie.dias@mrc.ac.za; 2 Division of Medical Physiology, Faculty of Health Sciences, Stellenbosch University, P.O. Box 19063, Tygerberg, Cape Town 7505, South Africa; 3 Department of Obstetrics and Gynecology, University of Pretoria, Private Bag X169, Pretoria 0001, South Africa; sumaiya.adam@up.ac.za

**Keywords:** intimate partner violence, stress, depression, pregnancy, insulin resistance, gestational diabetes mellitus

## Abstract

The early detection and management of gestational diabetes mellitus (GDM) is an important public health goal. GDM, which is defined as a glucose intolerance that develops during pregnancy, affects about 14% of pregnancies globally, and without effective treatment, it is associated with adverse short- and long-term maternal and neonatal outcomes. Risk-factor screening is an acceptable and affordable strategy to enable risk stratification and intervention. However, common biological risk factors such as overweight or obesity, excessive gestational weight gain, and family history of diabetes often have poor predictive ability, failing to identify a large proportion of women at risk of developing GDM. Accumulating evidence implicate psychosocial factors in contributing to GDM risk. As such, intimate partner violence (IPV), through its contributing effects on maternal stress and depression, presents a plausible risk factor for GDM. Experiencing IPV during pregnancy may dysregulate the hypothalamus–pituitary–adrenal (HPA) axis, leading to increased cortisol secretion and insulin resistance. These effects may exacerbate the insulin-resistant environment characteristic of pregnancy, thus increasing GDM risk. This review explores the relationship between IPV and GDM. We highlight studies that have linked IPV with GDM and propose a biological mechanism that connects IPV and GDM. Recommendations for IPV screening strategies to prevent GDM are discussed.

## 1. Introduction

Gestational diabetes mellitus (GDM) is defined as glucose intolerance that develops during pregnancy with glucose homeostasis usually restored after birth [1]. The prevalence of GDM has steadily increased over the last 20 years. Globally, ≈16.2% (21.3 million) of live births are associated with hyperglycemia in pregnancy, of which 86.4% are due to GDM, 6.2% are due to pre-existing type 1 diabetes (T1D) or type 2 diabetes (T2D), and 7.4% are due to T1D and T2D first detected during pregnancy [2]. Without appropriate glucose management, GDM is associated with increased perinatal complications and risk of future metabolic disease in both mother and child [3,4,5]. Many international bodies advocate for the universal screening and detection of GDM as a strategy to reduce the delay of diagnosis and improve pregnancy outcomes [6]. However, this is not adhered to globally, particularly in low- and middle-income countries. The high costs and technical challenges of the oral glucose tolerance test (OGTT), the gold standard for GDM diagnosis, has resulted in many countries using risk factor-based selective screening as the cornerstone of GDM diagnosis. However, traditional risk factors have poor predictive value and fail to identify a large percentage of women at risk of developing GDM, thus limiting their use [6]. It is thus clear that there are other yet unidentified risk factors that may be associated with the development of GDM. The identification of additional risk factors may improve the diagnosis and detection of GDM and therefore, it is a public health priority. Accumulating evidence implicate behavioral and psychosocial factors in contributing to GDM risk, suggesting that these factors should be incorporated into risk stratification models, which are currently mainly based on biological risk factors. 

Intimate partner violence (IPV) is a public health crisis that affects between 2% and 13.5% of pregnancies globally, depending on the population studied and the diagnostic method used [7]. More recently, rates as high as 27% and 42% have been reported in Brazil [8] and South Africa [9], respectively, highlighting the global crisis. IPV is associated with maternal and fetal morbidity and mortality, either through direct effects on the pregnancy or via indirectly affecting maternal biological pathways [10,11]. IPV-induced depression and stress leads to hyperactivity and dysregulation of the hypothalamus–pituitary–adrenal (HPA) axis, increased cortisol secretion, and the development of insulin resistance [12]. Given the high prevalence of IPV and the rising rates of GDM, it is important to understand the relationship between these two conditions to potentially reduce adverse pregnancy outcomes in this high-risk population. The combined effects of insulin resistance due to pregnancy and IPV may act synergistically to increase the risk of developing GDM [13], thus identifying IPV as a plausible risk factor for GDM. In this short narrative review, we explore the relationship between IPV and GDM. A PubMed literature search using the keywords “intimate partner violence”, “abuse”, “domestic violence”, and “gestational diabetes” was performed to identify studies on IPV and GDM. We highlight studies that have linked IPV with GDM and propose a biological mechanism through which IPV acts as a precipitating insult for the development of GDM. We hypothesize that during pregnancy, IPV-induced depression and stress exacerbates insulin resistance in metabolically active tissues leading to pancreatic beta (β) cell dysfunction and GDM. Therefore, IPV presents a risk factor that could be targeted to prompt GDM diagnosis. Recommendations for IPV screening to improve the diagnosis of GDM are discussed. 

## 2. Pregnancy: An Insulin-Resistant State

**Figure 1:**
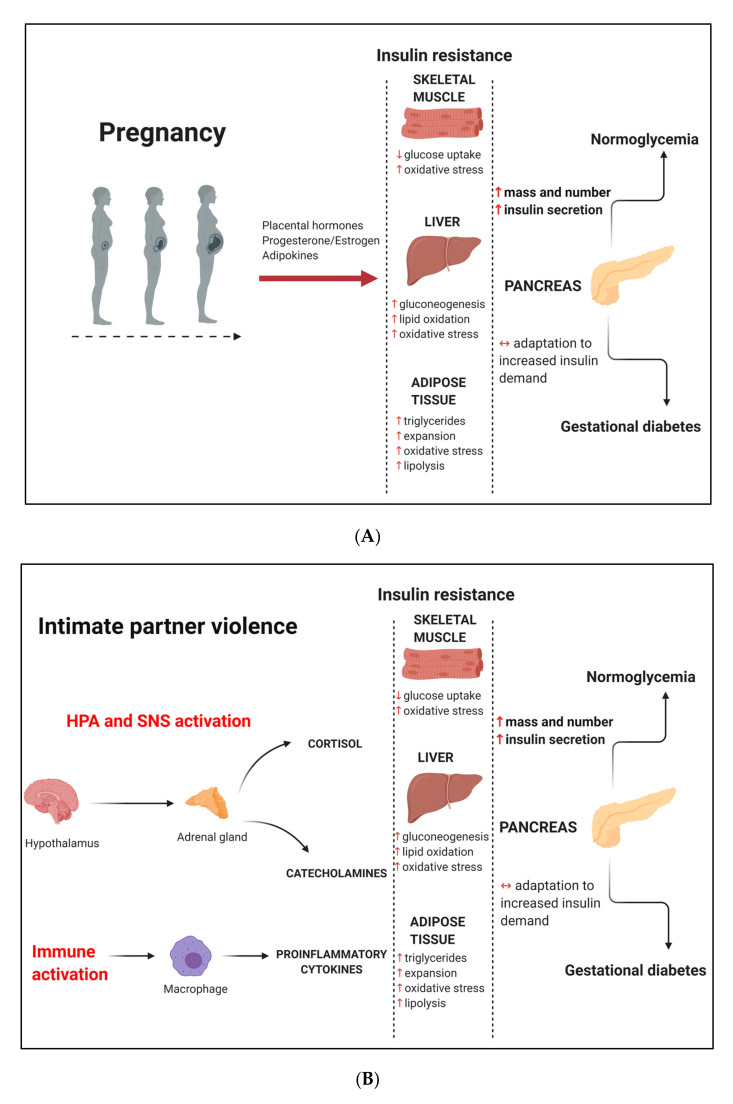

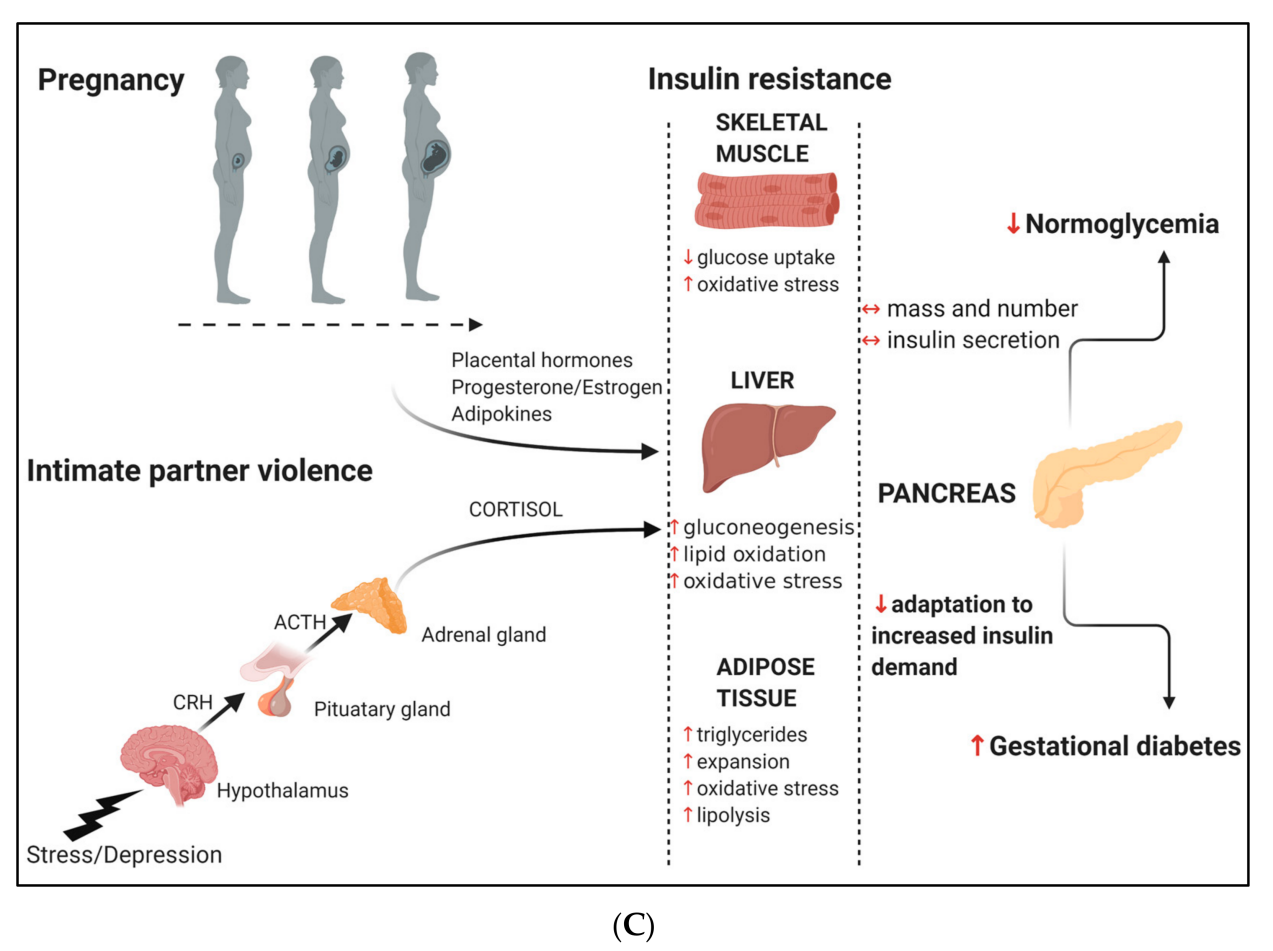
The effect of pregnancy and intimate partner violence (IPV) on the development of gestational diabetes mellitus (GDM). (**A**). Pregnancy-induced insulin resistance leads to the development of GDM in women who are not able to mount a compensatory β-cell response. (**B**). IPV-induced stress and depression leads to insulin resistance and an increased GDM risk. (**C**). Pregnancy- and IPV-induced insulin resistance that occur concurrently decreases the compensatory ability of pancreatic beta cells to increase insulin production and secretion, leading to an increased risk of developing GDM. Abbreviations: ACTH, adrenocorticotropic hormone; CRH, corticotropin-releasing hormone; HPA, hypothalamus–pituitary–adrenal; SNS, sympathetic nervous system. ↑ increase, ↓ decrease, ↔ unable to adapt (Image created with Biorender.com).

Pregnancy is characterized by insulin resistance due to several physiological, biochemical, and metabolic adaptations to meet the changing nutritional needs of the developing fetus [14,15,16]. The first phase of pregnancy is an anabolic process characterized by increased de novo lipogenesis in the liver. Later in gestation, peaking in the third trimester, pregnancy enters a catabolic phase where these lipids are preferentially broken down for maternal use, sparing glucose to meet the increasing energy demands of the growing fetus [15,16,17]. These normal pregnancy adaptations are associated with decreased insulin sensitivity and increased lipid oxidation and oxidative stress in skeletal muscle, decreased insulin sensitivity and increased gluconeogenesis, lipid oxidation and oxidative stress in the liver, and decreased insulin sensitivity and increased triglycerides, tissue expansion, lipolysis, and oxidative stress in adipose tissue [15] (Figure 1A). Decreased insulin sensitivity is a critical metabolic adaptation during pregnancy, allowing the shunting of glucose to the fetus to promote development while maintaining adequate maternal nutrition [18]. The term “insulin resistance” is defined as a pathological condition where metabolically active tissues do not respond to normal physiological concentrations of insulin, leading to glucose intolerance due to impaired insulin-receptor signaling [19]. To compensate for insulin resistance, pancreatic β-cells use several adaptive mechanisms, including increasing mass, number, and insulin-secretory capacity to maintain glucose homeostasis [15]. It has been estimated that maternal insulin secretion is increased up to 250% in order to maintain normoglycemia during pregnancy and that insulin-stimulated glucose disposal is decreased by about 40–60% during this time [20,21]. When insulin production does not overcome insulin resistance, possibly due to β-cell dysfunction, glucose intolerance and hyperglycemia occurs [22,23]. The underlying mechanisms for the development of insulin resistance during pregnancy are not yet fully elucidated, although several lines of evidence from both in vitro and in vivo studies show that various tissues including the placenta, ovaries, and adipose tissue contribute to the state of insulin resistance. Placental hormones such as human placental lactogen, leptin, and placental growth hormone upregulate the growth hormone/insulin-like growth factor axis to promote nutrient transfer to the fetus and enable fetal growth [24]. In addition, increased levels of estrogen, progesterone, and adipocyte-derived hormones such as adiponectin, resistin, tumor necrosis factor alpha (TNFα), interleukin-6 (IL6), and C-reactive protein (CRP) are also suggested to play a role in the development of insulin resistance during pregnancy [14,20,24,25,26]. 

## 3. Gestational Diabetes Mellitus 

Gestational diabetes mellitus (GDM) is a common metabolic disorder that develops in pregnant women who are unable to mount a compensatory β-cell response to counteract insulin resistance, thus leading to glucose intolerance [27]. It has been estimated that insulin sensitivity is decreased by up to 40% in pregnant women with GDM compared to women with normoglycemia [21,28]. Although GDM is reported to affect ≈14% of pregnancies globally, rates between 1% and 28% are reported in different regions, varying according to ethnicity, geographical location (urban vs. rural), environmental factors, and the screening and diagnostic strategies employed [29]. GDM imposes significant economic costs on health systems. The health care costs of treating women with GDM are ≈25.1% higher than treating women without GDM [30]. In 2007, it was estimated to cost $636 million to treat GDM ($596 million for maternal costs and $40 million for neonatal costs) in the USA [31]. These costs may be considerably more today given the rising prevalence of GDM. GDM is associated with serious short- and long-term complications for both mothers and their children [3,4,5]. It is associated with maternal (pre-eclampsia, increase in caesarean deliveries, birth trauma), fetal (macrosomia, hypoglycemia, shoulder dystocia), and perinatal (respiratory distress syndrome, jaundice, metabolic derangements) complications and is one of the leading causes of morbidity and mortality for mothers and infants worldwide. Women with GDM have an increased risk of developing obesity, metabolic disease, and type 2 diabetes in later life [3]. Furthermore, the “*developmental origins of health and disease* (DOHaD)” theory proposes intergenerational consequences where the offspring of mothers with GDM have an increased risk of obesity and metabolic syndrome during childhood and later in life [32]. 

The screening and diagnosis of GDM is contentious, with no uniform strategy adhered to globally [6]. Universal testing for GDM using the OGTT, conducted at 24–28 weeks of gestation, is widely advocated as a strategy to promote timely treatment and improve pregnancy outcomes [33], although its implementation varies across countries and institutions. Many countries screen for GDM using traditional risk factors, while in the USA, the glucose tolerance test that involves administering a 50 g glucose load to pregnant women at 24–28 weeks of gestation, irrespective of fasting is commonly used. Women who screen positive are referred for the OGTT, which involves administration of a 75 g glucose load to pregnant women in a fasted state. The recommendations for universal testing are based on findings from the Hyperglycemia and Adverse Pregnancy Outcome (HAPO) study. The HAPO study, which involved 23,316 pregnant women across nine countries, showed that mild levels of glucose intolerance, even at glucose concentrations previously considered normal, are associated with adverse pregnancy outcomes, highlighting the urgent need for early detection and prompt treatment [34]. However, the recommendation for universal testing is not adhered to globally, particularly in low-and middle-income countries. The high costs and technical challenges of the OGTT have resulted in many countries using risk factor-based selective screening, followed by OGTT diagnosis, as the cornerstone of GDM diagnosis [6]. Unfortunately, the currently available risk factors (overweight or obesity, excessive gestational weight gain, advanced maternal age, family history of diabetes, previous history of GDM, and adverse pregnancy outcomes) have poor predictive value and fail to identify a large percentage of women with GDM, thus limiting their use as screening tools [29,35,36]. Therefore, the identification of novel risk factors or biomarkers that could be targeted to prompt OGTT screening and GDM diagnosis has become an increasing focus of GDM research. Furthermore, the earlier detection of women at risk for GDM would facilitate the earlier initiation of treatment, which could markedly improve pregnancy outcomes for both mother and child. In recent years, the search for risk factors have focused on investigating the potential of biological factors such as maternal serum proteins including adiponectin, insulin, sex hormone binding globulin, C-reactive protein, and glycosylated fibronectin, and genetic (single nucleotide polymorphisms) and epigenetic (DNA methylation and microRNAs) markers to serve as biomarkers for GDM [37]. However, these potential biomarkers are yet to achieve clinical applicability, underscoring the complex pathophysiology of GDM. Furthermore, it is still not clear why some pregnant women develop GDM, while others do not, although underlying insulin resistance and genetic susceptibility have been suggested to play a role in disease development [29]. In recent years, accumulating evidence implicate behavioral and psychosocial factors, in addition to the traditional biological factors described previously, in the development of metabolic diseases including GDM [38,39,40].

## 4. Intimate Partner Violence

Intimate partner violence (IPV) is defined as a form of abuse within an intimate relationship (married, unmarried, or live-in) that causes physical, psychological, or sexual harm to those in that relationship [41]. This definition encompasses physical, sexual, and psychological aggression/abuse or controlling behavior of any kind. IPV is a human rights violation and an important public health crisis with significant health and economic costs [42]. Various cultural, political, legal, and economic factors perpetuate IPV, often with devastating consequences for the abused and negatively impacting the wider society [43]. Population-based studies have identified young age, low socioeconomic status, fair or poor mental health of victims and/or their partners, low education level, drug or alcohol abuse, high number of sexual partners, poor social support, and threat or abuse of pets as risk factors for IPV [43,44]. Although both men and women may be perpetrators, women comprise the majority of IPV victims and incur the most serious IPV-related injury [45]. IPV is the most common form of violence that women experience. Globally, it is estimated that 30% of women aged 15 years have experienced IPV during their lifetime, ranging between 16% to 66% across regions [41], which is probably due to differences in diagnostic methods and socioeconomic conditions. Unfortunately, pregnant women are not spared from this form of abuse. During pregnancy, women are especially vulnerable to IPV due to an increase in their physical, social, emotional, and economic needs. A study measuring IPV prevalence across 19 countries reported that rates varied between 2.0% and 13.5% depending on the population studied, contextual factors, and the instruments used to assess IPV [7,46,47,48]. Alarmingly, up to 27% and 42% of pregnant women in South Africa [9] and Brazil [8], respectively reported that they were victims of IPV. Psychological abuse is the most common form of IPV experienced during pregnancy. A meta-analysis of risk factors for domestic violence during pregnancy reported that the average prevalence of emotional abuse was 28.4%, physical abuse was 13.8%, and sexual abuse was 8.0% [49]. IPV negatively impacts mothers and their unborn babies and has been linked to detrimental physical and mental health outcomes for both [48]. The most severe effect of IPV during pregnancy results from physical assaults, which in extreme cases may lead to death of the mother and/or the fetus [46]. During pregnancy, women who are abused may be at a three-fold higher risk of femicide compared to women who are not abused [46]; therefore, abuse during pregnancy is an important risk factor for attempted or completed femicide. Many studies have provided evidence of the impact of IPV on adverse pregnancy outcomes (Supplementary Table S1, Supplementary Figure S1), with a recent systematic review of 50 studies conducted in Asia, North America, South America, Africa, and Europe, reporting that the most common adverse outcomes associated with IPV during pregnancy are preterm birth, low birthweight, miscarriage, perinatal death, and premature rupture of membranes [50]. Maternal adverse effects include physical morbidity, low maternal weight gain, kidney infections, urinary tract infections, vaginal infections, and caesarian delivery [10]. Despite the vast body of literature on the effects of IPV on pregnancy outcomes, our literature search identified no studies linking IPV to maternal metabolic health during pregnancy. Associations between IPV and risk for metabolic disorders in women have been reported in previous studies, although not during pregnancy (Table 1). IPV has been associated with various markers of cardiometabolic disease including abdominal obesity, low high-density lipoprotein cholesterol, elevated triglycerides, type 2 diabetes, and hypertension [51,52,53,54,55]. These results highlight the importance of considering IPV as a risk factor for metabolic disease and the need to elucidate the underlying mechanisms that link IPV and metabolic dysregulation.

Stress and depression promote metabolic dysregulation through the stimulation of pro-inflammatory responses and activation of neuroendocrine pathways, which produce stress hormones that induce insulin resistance and dysregulated glucose metabolism [56,57,58] (Figure 1B). The stimulation of insulin resistance and hyperglycemia is an adaptive stress response to enable adequate energy requirements for the fight-or-flight response [59]. During conditions of stress, pro-inflammatory genes in immune cells are stimulated, thereby increasing the production of pro-inflammatory cytokines [57]. The relationship between inflammation and insulin resistance has been well described [60,61]. Neuroendocrine pathways are the most widely and comprehensively studied potential mediators between stress metabolic dysregulation [58]. The HPA axis and the sympathetic nervous system (SNS) were the first neuroendocrine systems shown to be closely related to stress. During conditions of stress, the hypothalamus is activated to produce the corticotropin-releasing hormone (CRH). CRH is transported through the blood and acts on the anterior pituitary to activate corticotroph cells to secrete adrenocorticotrophin hormone (ACTH) into the general circulation. ACTH travels through the blood to the adrenal cortex and promotes the synthesis of the glucocorticoid cortisol the primary stress hormone [62]. Stress also activates the SNS, thereby stimulating the release of neurotransmitters or catecholamines such as norepinephrine and adrenaline from the adrenal medulla. Corticosteroids and catecholamines promote insulin resistance in peripheral tissues. Although cortisol concentrations are regulated by a negative feedback mechanism acting at both the anterior pituitary and within the hypothalamus to inhibit the further release of ACTH and CRH, respectively, conditions of metabolic imbalance lead to further activation of the hypothalamus. The HPA axis is arguably the most studied neuroendocrine pathway and the focus of this review. Dysregulation of the HPA axis presents a plausible mechanism that may link IPV with metabolic dysregulation and adverse pregnancy outcomes. These effects may be mediated by the actions of IPV-induced stress and depression on activation of the HPA axis, leading to increased cortisol secretion, which is often used as a biomarker of HPA axis activity [11,12,63]. 

## 5. Association between IPV and GDM 

The importance of cardiometabolic health during pregnancy is widely recognized. As such, women are routinely screened for common pregnancy-related disorders such as GDM and hypertension, although behavioral and psychological factors are often neglected. A study of sociodemographic and behavioral risk factors and the most common medical conditions during pregnancy (diabetes, hypertension, preeclampsia, and body mass index (BMI)) in a population of high-risk, urban, African American mothers demonstrated that IPV was the 4th strongest predictor of poor pregnancy outcomes after prepregnancy BMI, preconceptional diabetes, and employment status [64]. GDM was also associated with these adverse pregnancy outcomes, albeit not as strongly. Another USA-based study found that IPV, but not GDM, was significantly associated with pregnancy trauma and placenta abruption [65]. IPV and GDM share risk factors such as low socioeconomic status and low education level [29,44], suggesting that IPV and GDM may have a shared etiology and could interact to increase disease severity. Therefore, we conducted a literature search to identify studies linking these two conditions. We used keywords “intimate partner violence” OR “abuse” OR “domestic violence” AND “gestational diabetes” to increase the sensitivity of the search. The studies that reported on these conditions are summarized in Table 2 (Supplementary Figure S2). Both IPV and GDM are independent predictors of adverse pregnancy outcomes and pose significant economic costs to the health system [42], while their interaction may exacerbate these effects. IPV induces stress and depression [11,63,66,67], which are both associated with an increased risk for GDM [11,68,69]. Thus, stress and depression may act as mediators between IPV and GDM. Furthermore, abuse during childhood and adolescence has been linked with stress and depression [12,70], IPV [39], and as reported in Table 2, an increased likelihood of developing GDM [38,39,40] during adulthood. Evidence for biological mechanisms that link IPV and GDM is lacking, although it is conceptually plausible that these conditions may act synergistically to increase disease severity. Understanding the relationship between these conditions is important, particularly given the high prevalence of IPV among women of reproductive age and its roles as a potential risk factor for GDM. Stress and depression are common mental health consequences of IPV, which may present an additional mechanism of insulin resistance and glucose intolerance during pregnancy. IPV induces dysregulation of the HPA axis, leading to the hypersecretion of cortisol and insulin resistance. This insult may exacerbate the normal pregnancy insulin-resistant states, i.e., decreased insulin sensitivity and increased lipid oxidation and oxidative stress in skeletal muscle, decreased insulin sensitivity, and increased gluconeogenesis, lipid oxidation, and oxidative stress in liver and decreased insulin sensitivity and increased triglycerides, tissue expansion, lipolysis, and oxidative stress in adipose tissue [15]. These effects may place an additional burden on β-cells, which may lead to a reduced capacity to mount compensatory effects, leading to the development of GDM (Figure 1C). A higher prevalence of GDM was observed in women who reported IPV, however, this did not reach statistical significance [71]. Furthermore, associations between IPV and GDM are marked by inconsistent or unexpected results. Two studies failed to show an association between IPV and GDM [10,72] and may be due to variabilities in the populations studied or inadequate sensitivity and specificity of the psychometric instruments used [73], as highlighted in the study by Dahlen et al. [70]. Furthermore, most of the studies that investigated the association between psychological factors and GDM were retrospective or based on self-report, which is subject to bias, and lacked objective clinical measures of GDM. Despite the limited studies that have directly linked IPV with GDM it is apparent that screening for both these conditions should form part of postpartum care [74]. Large population-based studies are required to further explore the relationship between IPV and GDM.

## 6. Screening for IPV as a Risk Factor for GDM

Women are not screened for IPV during pregnancy, despite it possibly being more common than preeclampsia, placenta previa, and GDM, conditions for which women are routinely screened during prenatal visits [46]. Similar to these more familiar obstetric complications, IPV is associated with maternal and fetal morbidity and mortality, either through direct effects on the pregnancy or via depression or stress-induced insulin resistance and the development of GDM, as discussed previously [10]. We have proposed a biological mechanism linking GDM and IPV and suggest that IPV presents a risk factor for the development of GDM. Thus, IPV screening is an integral part of prenatal care, and it should be included in risk assessment algorithms to improve GDM detection to protect both mother and child [75]. Although IPV screening is notoriously challenging due to the woman’s feelings of embarrassment or fear of the partner finding out, prenatal visits may present an ideal opportunity to detect IPV to prevent GDM [76]. During pregnancy, the main concern of women is the well-being of their babies, and they may therefore be more likely to disclose sensitive information [77]. A study investigating the acceptability of IPV screening during pregnancy found that 97% of women screened reported that they were not embarrassed, angry, or offended when screened for the occurrence of IPV during pregnancy [78]. In addition, to the short-term benefits of screening for IPV to prevent obstetric complications, prenatal screening could also present a significant opportunity to decrease the morbidity and mortality associated with future IPV. Women who are victims of IPV during pregnancy are at risk of future IPV [79]. Universal screening for IPV and appropriate intervention during health care visits are widely advocated [13]. However, these recommendations are poorly adhered to, possibly due to the lack of effective screening and intervention programs. Barriers to IPV screening include the bias of health care workers with regard to who may be at risk, fear of offending the patient, powerlessness, lack of control over the situation or time constraints, and the poor sensitivity and specificity of current IPV screening tools [73]. Even well-established psychometric tests vary in sensitivity and specificity, while the lack of empirical data about the potential risks and benefits of screening further negatively impacts the implementation of screening strategies to decrease IPV. Education and training of health care workers are required to screen, identify, and refer women at risk to support services in a manner that portrays interest, caring, and comfort. The development of simple screening tools such as the R3 App (Recognize, Respond, and Refer), which refers to four simple questions related to Hurt, Insult, Threatening, and Screaming (HITS) [80], the identification of risk factors [81,82] and biomarkers for IPV during pregnancy [83] show potential. However, more research in diverse populations is required to develop sensitive screening tests that are universally acceptable and accessible. 

Motivation for the development of screening tools for IPV during pregnancy requires the availability of effective interventions that are easily implementable to minimize the harmful effects of IPV. IPV is associated with cultural and behavioral factors such as male identity and a woman’s gender role [43]. Therefore, screening for IPV should form part of an active collaboration with social support services that empower women and assist toward ending their victimization. Such initiatives require political commitment to guide policy and legislation targeted toward ending IPV. Primary IPV preventative strategies include creating a culture of non-tolerance of IPV, empowering women and improving their status in society, educating against violence as a means to address conflict, and facilitating research targeted to prevent IPV [41,43]. Critical to these strategies is economic development and health promotion to improve socioeconomic factors such as poverty, low levels of education, and alcohol abuse, which are factors that perpetuate IPV. To date, IPV has not been adequately incorporated in the political agenda. Until such time, health services must focus on educating women on healthy choices to decrease the impact of insulin resistance, which is a common mechanism in the pathophysiology of IPV and GDM. Prenatal care should include counseling women on healthy dietary choices and increasing physical activity or exercise, which are factors that improve insulin sensitivity [84]. Ideally, integrating and combining advice on modifiable factors such as diet, physical activity, and psychosocial well-being, which may be more effective than targeting these risk factors individually [85], should be encouraged. Functional foods such as polyphenols contain biologically active ingredients that show promise to protect against insulin resistance and GDM [86,87,88]. Due to the life-course effects of IPV and GDM during pregnancy, prevention of these conditions offers tremendous potential to improve pregnancy outcomes and maternal and child health.

## 7. Limitations

Although we conducted a wide-ranging search to identify studies investigating IPV and GDM, we may have missed some studies. The details of the search strategy are included as Supplementary Figures. To our knowledge, this is the first review to collate the available evidence on IPV and GDM, so although it may not be exhaustive, it represents the most comprehensive attempt to explore the relationship between these conditions and draws attention to this important research area. Another major limitation of this type of research is an underestimation of IPV due to a lack of universally appropriate screening tools to assess IPV [73]. Research initiatives to develop sensitive psychometric IPV screening tools that are acceptable to women and applicable to diverse populations are urgently needed. Furthermore, women may fear disclosing IPV due to fear of retaliation from their partners or the risk of having their babies removed by Child Protection Services if they reveal violence in the home [76]. Thus, strategies to support women to increase their safety and lessen harm are critical.

## 8. Conclusions

This review has proposed a biological mechanism whereby IPV during pregnancy can contribute to the development of GDM. IPV-induced stress and depression may lead to dysregulation of the HPA axis, leading to the hypersecretion of cortisol and the subsequent development of insulin resistance. These effects may exacerbate the insulin resistant environment characteristic of pregnancy, causing severe insulin resistance in skeletal muscle, liver, and adipose tissue, ultimately reducing the compensatory effects of pancreatic β-cells and leading to the development of GDM. Therefore, in addition to the direct physical and mental effects on the health of mother and child, we suggest that IPV during pregnancy contributes to the development of GDM. We suggest that IPV presents an additional risk factor for GDM and recommend that IPV screening should form part of prenatal visits so that effective interventions can be applied to mitigate the deleterious interactions between IPV and pregnancy to prevent the development of GDM. Large population-based studies are required to further explore our hypothesis and to make recommendations toward public health interventions to improve pregnancy outcomes. An integration of physical and mental health care during pregnancy is critical to improve the health and well-being of mothers and their children. 

## Figures and Tables

**Table 1:** Association between IPV and cardiometabolic disease.

Author	Study Details	Country	Sample Size (n)	Outcome	Estimate (95% CI)
[51]	A prospective population-based cohort study conducted between 2001 and 2007 to investigate the association between IPV and hypertension. Risk was measured using hazard ratios.	USA	51,434	**Hypertension** Physical IPV Sexual IPV Psychological IPV	1.1 (1.0;1.1) 1.0 (0.9;1.1) 1.2 (1.0;1.5)
[52]	A prospective population-based cohort study conducted between 2001 and 2007 to investigate the association between IPV and type 2 diabetes risk. Risk was measured using hazard ratios.	USA	64,732	**Type 2 diabetes** Physical IPV Psychological IPV	1.1 (0.9–1.3) 1.6 (1.1–2.4)
[53]	A retrospective cohort study of primary care patients conducted between 1995–2017 to investigate the association between IPV and cardiometabolic risk. Risk was measured using incidence rate ratios.	UK	18,547	Cardiovascular disease Hypertension Type 2 diabetes mellitus All-cause mortality	1.3 (1.1;1.6) 1.0 (0.9;1.1) 1.5 (1.3;1.8) 1.4 (1.2;1.7)
[54]	A prospective population-based cohort study conducted between 2005–2016 to investigate the association between IPV cardiometabolic risk. Risk was measured using hazard ratios.	USA	18,133	Type 2 diabetes Hypertension	1.7 (1.1;2.5) 1.0 (0.6;1.6)
[55]	A prospective population-based cohort study conducted between 2000–2002 to investigate the association between IPV and cardiometabolic risk. Risk was measured using incidence rate ratios.	Norway	5593	**Increased metabolic risk** Abdominal obesity, low high-density lipoprotein cholesterol, and elevated triglycerides. Antihypertensive medication	1.4 (1.1;1.7)

Abbreviations: CI, confidence interval; IPV, intimate partner violence; UK, United Kingdom; USA, United States of America.

**Table 2:** Studies that have reported on GDM and IPV/abuse.

Author	Study Description	Country	Sample Size (n)	Outcome	Estimate(95% CI)
**Sociodemographic, Behavioral and Psychosocial Risk Factors**					
[10]	A population-based study to investigate the association between IPV and adverse pregnancy outcomes.	Spain	779	No association between IPV and GDM was observed.	NE
[30]	A systematic review investigating the economic burden of common health problems associated with pregnancy and childbirth. Of the 38 studies included in the review 16 focused on GDM and 1 on IPV	USA, Ireland, Finland, Sweden, Italy, Brazil, South Korea China (GDM) USA (IPV)	GDM 3,027,237 IPV 32,658,259	**Costs per case** GDM IPV	€263-€13,680 €1,410
[64]	Population-based prospective study of high-risk, urban, African American mothers to investigate the association between biomedical, psychosocial and behavioral risks and adverse pregnancy outcomes.	USA	918	Predictor of poor pregnancy outcome IPV GDM	4th 17th
[65]	Population-based, retrospective cross-sectional study conducted to examine the association between IPV and pregnancy outcomes. Risk was measured using odds ratios.	USA	2,873	**IPV** Pregnancy trauma Placental abruption (odds ratio) **GDM**	14.4 (8.1;25.9) 4.0 (1.5;10.5) NE
[70]	A population-based cross-sectional study to investigate the association between psychosocial risk factors and pregnancy and birth outcomes. Risk was measured using risk ratios.	Australia	3,092	Psychosocial issues IPV risk ratio GDM risk ratio	7.3 (4.2;12.7) 1.9 (1.1;3.0)
[71]	A retrospective population-based study to investigate the association between IPV and pregnancy outcomes.	Australia	33, 542	GDM prevalence higher in women who reported IPV although statistically significant.	9.4% vs. 8.6%
[72]	Retrospective cross-sectional study conducted to investigate the association between sociodemographic and behavioral factors and risk of developing GDM.	USA	4,682	No association between abuse and GDM was observed.	NE
[74]	A review describing the importance of postpartum care.	USA	NA	**Recommendations towards postpartum care:** GDM screening Biopsychosocial assessment (e.g., depression, IPV)	NA
**Abuse during childhood or adolescence**					
[38]	A population-based cohort study conducted between 1996-2015 to investigate the association between childhood adverse events and risk of developing GDM. Risk was measured using risk ratios.	Australia	6,317	GDM in women with preconception depressive symptoms. Three adverse childhood events Four adverse child events	1.7 (1.0;3.0) 1.8 (1.0;3.0)
[39]	A population-based longitudinal cohort study of the Nurses’ Health Study II to determine whether childhood abuse is associated with risk of autism, and other pregnancy outcomes including GDM.	USA	52,949	GDM prevalence higher in women exposed to the highest level of abuse compared to women who were not exposed to abuse.	5.3% vs. 2.7%
[40]	A retrospective cohort study conducted in the Nurses’ Health Study II to investigate whether childhood or adolescent abuse victimization is associated with a risk of developing GDM. Risk was measured using risk ratios.	USA	45,550	GDM Severe physical abuse Forced sexual activity	1.4 (1.2;1.7) 1.3 (1.1;1.5)

Abbreviations: CI, confidence interval; GDM, gestational diabetes; IPV, intimate partner violence; NA, not applicable; NE, no effect; USA, United States of Americ.

## Supplementary Materials

The following are available online at https://www.mdpi.com/1660-4601/17/21/7843/s1, Figure S1: Flow chart for selection of studies reporting on IPV and pregnancy outcomes used in Table S1, Figure S2: Flow chart for selection of studies reporting on IPV/abuse and GDM used in Table 2, Table S1. Association between IPV and pregnancy outcomes.
