# Supplementary material for: Intimate Partner Violence: A Risk Factor for Gestational Diabetes
Source: Int J Environ Res Public Health. 2020 Oct 26;17(21):7843. doi: 10.3390/ijerph17217843 (PMC7663316; doi:10.3390/ijerph17217843)
Supplement: Supplementary file 1 [file ijerph-17-07843-s001.pdf]

Supplementary Material

Review

Intimate Partner Violence: A Risk Factor for Gestational Diabetes

Carmen Pheiffer [1,2,*], Stephanie Dias [1] and Sumaiya Adam [3]

[1] Biomedical Research and Innovation Platform (BRIP), South African Medical Research Council, P.O. Box 19070, Tygerberg, Cape Town 7505, South Africa; stephanie.dias@mrc.ac.za

[2] Division of Medical Physiology, Faculty of Health Sciences, Stellenbosch University, P.O. Box 19063, Tygerberg, Cape Town 7505, South Africa

[3] Department of Obstetrics and Gynecology, University of Pretoria, Private Bag X169, Pretoria 0001, South Africa; sumaiya.adam@up.ac.za

* Correspondence: carmen.pheiffer@mrc.ac.za; Tel.: (+27 21 938 0292)

Table S1. Association between IPV and pregnancy outcomes.

Author	Country	Sample size (n)	Outcome	ODDS (95% CI)
(Gebreslasie et al., 2020)	Ethiopia	648	Still birth	3.3 (1.1;9.7)
(FitzPatrick et al., 2020)	Australia	1,507	Depressive symptoms<br>Self-reported anxiety	4.6 (2.9;7.1)[a]<br>2.9 (1.9;4.4)[a]
(Tiruye et al., 2020)	Ethiopia	4,167	Pregnancy loss	1.5 (1.1;2.1)[a]
(Kaiyo-Utete et al., 2020)	Zimbabwe	375	Depression	2.5 (1.5;4.2)
(Finnbogadóttir et al., 2020)	Sweden	1,939	Caesarian section	1.3 (1.0;1.7)
(Coker et al., 2004)	USA	755	Perinatal death<br>Preterm low birthweight<br>Low birthweight	2.1 (1.3;3.4)[b]<br>2.4 (1.5;4.0)[b]<br>1.9 (1.0;3.4)[b]
(Hill et al., 2016)	**Meta-analysis**<br>19 studies conducted in Asia (4), North America (10), South America (1), Europe (3) and Australasia (1)		Low birthweight<br>Preterm birth	1.2 (1.1;1.3)<br>1.4 (1.2;1.6)
(Gebrekristos et al., 2020)	South Africa	61	Sexually transmitted infection	4.4 (1.3;15.0)[b]
(Pastor-Moreno et al., 2020a)	**Meta-analysis**<br>50 studies conducted in Asia (15), North America (12), South America (12), Africa (6) and Europe (5)<br>The most frequent outcomes reported were pre-term birth (50%), low birthweight<br>(46%), miscarriage (30%), perinatal death (20%) and premature<br>rupture of membranes (20%)		**All types of IPV**<br>Preterm birth<br>Low birthweight<br>Miscarriage<br>Perinatal death<br>Premature rupture of membranes<br>Small gestational age<br>**Physical IPV**<br>Pre-eclampsia<br>Haemorrhage	**ODDS**<br>2.0-6.0<br>2.1<br>1.7–5.4<br>1.9- 5.2<br>3.2<br>2.4-4.0<br><br>2.7<br>1.9-8.6
(Field et al., 2018)	South Africa	376	Mental health problems	1.3 (1.3;2.7)[a]
(Habtamu Belete et al., 2019)	Ethiopia	342	Antenatal depression	4.5 (1.3;15.5)[a]
(Pastor-Moreno et al., 2020b)	**Meta-analysis**<br>17 studies conducted in Asia (5),		**Infant perinatal death**<br>Unspecified IPV	 3.2 (1.9;5.4)

	North America (5),<br>South America (4) and Africa (3)		Physical IPV<br>Any type of IPV	2.5 (1.8;3.4)<br>2.9 (2.0;4.1)
(Belay et al., 2019)	Ethiopia	589	Depression	17.6 (6.2;50.1)[a]
(Martin-de-las-Heras et al., 2019)	Spain	779	**Psychological IPV**<br>Urinary tract infection<br>Vaginal infection<br>Spontaneous preterm labour<br>**Physical IPV**<br>Antenatal hospitalizations	 1.9 (1.2;3.0)[a]<br>2.4 (1.2;4.7)[a]<br>2.2 (1.1;4.5)[a]<br><br>2.6 (1.0;7.1)[a]
(Tho Nhi et al., 2019)	Vietnam	1,337	**Postpartum depression**<br>Physical violence<br>Sexual violence	 2.8 (1.2;6.4)[a]<br>1.9 (1.0;3.7)[a]
(Khan et al., 2019)	Honduras, the Dominican Republic, Colombia, Peru, and Guatemala	19,563,610	Pregnancy termination	1.4 (1.3;1.5)
(Martin-de-Las-Heras et al., 2019a)	Spain	779	**Poor antenatal clinic attendance** Physical IPV<br>Psychological IPV	3.3 (1.1;9.4)[a]<br>1.6 (0.9;3.1)[a]
(Thomas et al., 2019)	USA	930	Depression<br>Anxiety<br>Prenatal distress	3.0 (1.6;5.7)[a]<br>2.2 (1.0;4.8)[a]<br>2.6 (1.3;5.1)[a]
(Jaraba and Garcés-Palacio, 2019)	Colombia	14,520	Preterm birth	2.0 (1.2;3.2)
(Komori et al., 2019)	Japan	79,985	Newborn hearing screening referral	1.4 (1.1;2.0)[a]
(Berhanie et al., 2019)	Ethiopia	954	Low birth weight<br>Preterm birth	3.1 (1.5;6.6)[a]<br>2.5 (2.2;3.0)[a]
(Martin-de-Las-Heras et al., 2019b)	Spain	779	Breastfeeding avoidance	2.0 (1.2;3.3)[a]
(Yu et al., 2018)	China	900	Prenatal depression<br>Adverse birth outcome[c]	2.5 (1.6;3.9)[a]<br>1.7 (1.1;2.6)[a]
(Jamieson, 2020)	**Metanalysis**<br>30 studies conducted in Asia (7),<br>North America (16),<br>South America (4), Europe (1) and Australasia (2)		Never enter care<br>Delay care	2.6 (1.6;4.4)<br>1.8 (1.5;2.2)

(Rurangirwa et al., 2018)	Rwanda	921	**Post-traumatic stress disorder**	
			Physical IPV	4.5 (2.1;9.6)[a]
			Psychological IPV	6.3 (3.2;12.5)[a]
			Sexual IPV	6.2 (3.0;12.9)[a]
			**Suicidal ideation**	
			Physical IPV	3.2 (1.5; 6.2)[a]
			Psychological IPV	3.0 (1.4; 6.6)[a]
			Sexual IPV	2.9 (1.5;5.6)[a]
(Shamu et al., 2018)	Zimbabwe	2,042	**Emotional IPV**	
			Low birth weight	1.8 (1.3;2.5)[a]
			Low birth weight/preterm birth/emergency caesarean section	1.4 (1.0;1.8)[a]
(Dahlen et al., 2018)	Australia	33,542	Threatened preterm labour	1.8 (1.3;2.4)[a]
(Ferdos et al., 2018)	Bangladesh	400	Medical complications	2.1 (1.2;-4.0)[a]
			Obstetric complications	4.2 (2.0;7.1)[a]
			Any pregnancy complication	5.3 (3.0;10.5)[a]
(Singh et al., 2018)	Nepal	426	Register for antenatal care	0.3 (0.1;0.5)
(Miura and Fujiwara, 2017)	Japan	6,590	**Postpartum depression**	
			Verbally IPV	4.9 (2.2;10.6)
			Physically IPV	7.1 (2.8;18.0)
(Islam et al., 2017b)	Bangladesh	426	**Postpartum depression**	
			Physical IPV	1.8 (1.3;3.4)[a]
			Sexual IPV	2.3 (1.1;4.5)[a]
			Psychological IPV	6.9 (1.7;28.0)[a]
(Suzuki et al., 2018)	Japan	470	Depression	5.0 (2.0-12.0)
			Anxiety	7.4 (2.7-20.0)
			Depression and anxiety	7.3 (2.9-18.0)
(Islam et al., 2017a)	Bangladeshi	426	**Physical IPV**	
			Delayed entry into prenatal care	2.6 (1.3;5.1)
(Sigalla et al., 2017)	Tanzania	1112	**Physical IPV**	
			Preterm birth	2.9 (1.3;6.5)[a]
			Low birth weight	3.2 (1.3;7.7)[a]
(Rogathi et al., 2017)	Tanzania	1013	Post-partum depression	3.1 (2.0;4.4)[a]
(Ogunwale et al., 2017)	USA	613	Initiation of prenatal care<br>Term delivery<br>Normal birth weight	0.4 (0.2;0.8)[a]

(Hoang et al., 2016)	Vietnam	1276	**Physical IPV**<br>Preterm birth<br>Low birth weight	 5.5 (2.1;14.1)<br>5.7 (2.2;14.9)
(Donovan et al., 2016)	**Meta-analysis**<br>50 studies conducted in North America (23), Asia (8), Africa (6), Europe (4), South America (6), Australasia (3)		Preterm birth<br>Low birth weight<br>Small gestational age	1.9 (1.6;2.3)<br>2.1 (1.7;2.7)<br>1.4 (1.0;1.8)
(Laelago et al., 2017)	Ethiopia	183	Low birth weight	14.3 (5.0;40.7)[a]
(Demelash et al., 2015)	Ethiopia	387	Low birth weight	3.0 (1.6; 7.2)
(Abdollahi et al., 2015)	Iran	1461	Low birth weight<br>Premature rapture of membrane	2.9 (1.9;4.4)<br>1.9 (1.1;3.1)
(Alhusen et al., 2014)	USA	239	Small gestational age<br>Low birth weight	4.8 (1.9;12.5)<br>4.2 (1.5;12.1)
(Hassan et al., 2014)	Iran	1,300	Preterm labour<br>Caesarean section<br>Antenatal hospitalization<br>Vaginal bleeding	1.5 (1.2;2.0)[a]<br>11.8 (6.4;22.0)[a]<br>6.3 (3.8;10.5)[a]<br>1.5 (0.9;2.3)[a]
(Han and Stewart, 2014)	**Review**<br>31 studies conducted in South America (Peru, Bolivia, Colombia, Brazil, Mexico, Guatemala, Costa Rica, Nicaragua, Haiti and Dominican Republic)		Maternal depression, pregnancy-related symptom distress, inadequate prenatal care, vaginal bleeding, spontaneous abortion, gestational weight gain, high maternal cortisol, hypertension, pre-eclampsia, sexually transmitted disease) and infant prematurity, low birth weight, neonatal complications and stillbirth	Significant association
(Mahenge et al., 2013)	Tanzania	1,180	Post-traumatic stress disorder Anxiety<br>Depressive	2.9 (1.7;5.1)[a]<br>4.0 (2.9;5.6)[a]<br>3.3 (2.4;4.6)[a]
(Sanchez et al., 2013)	Peru	959	Preterm labour	2.0 (1.5;2.6)[a]
(Stöckl et al., 2012)	Germany	401	Physical IPV<br>Pregnancy loss	8.3 (2.0;34.6)
(Ludermir et al., 2010)	Brazil	1,045	Postnatal depression	3.3 (2.3;4.7)
(Leone et al., 2010)	USA	2,873	Pregnancy trauma<br>Placental abruption	32.1 (14.3;71.8)[a]<br>5.2 (1.4;19.5)[a]

(Fanslow et al., 2008)	New Zealand	2,218	Spontaneous abortion<br>Termination of pregnancy	1.4 (1.1;1.8)<br>2.5 (1.8;3.4)
(Janssen et al., 2003)	Canada	4,750	**Physical violence**<br>Antepartum hemorrhage Intrauterine growth restriction Perinatal death	3.8 (1.4;10.4)[a]<br>3.1 (1.0;9.1)[a]<br>8.1 (1.4;45.6)[a]

[a] Adjusted ODDS [b] Adjusted Risk ratio [c]Preterm birth, low birth weight, birth defects, asphyxia and stillbirth

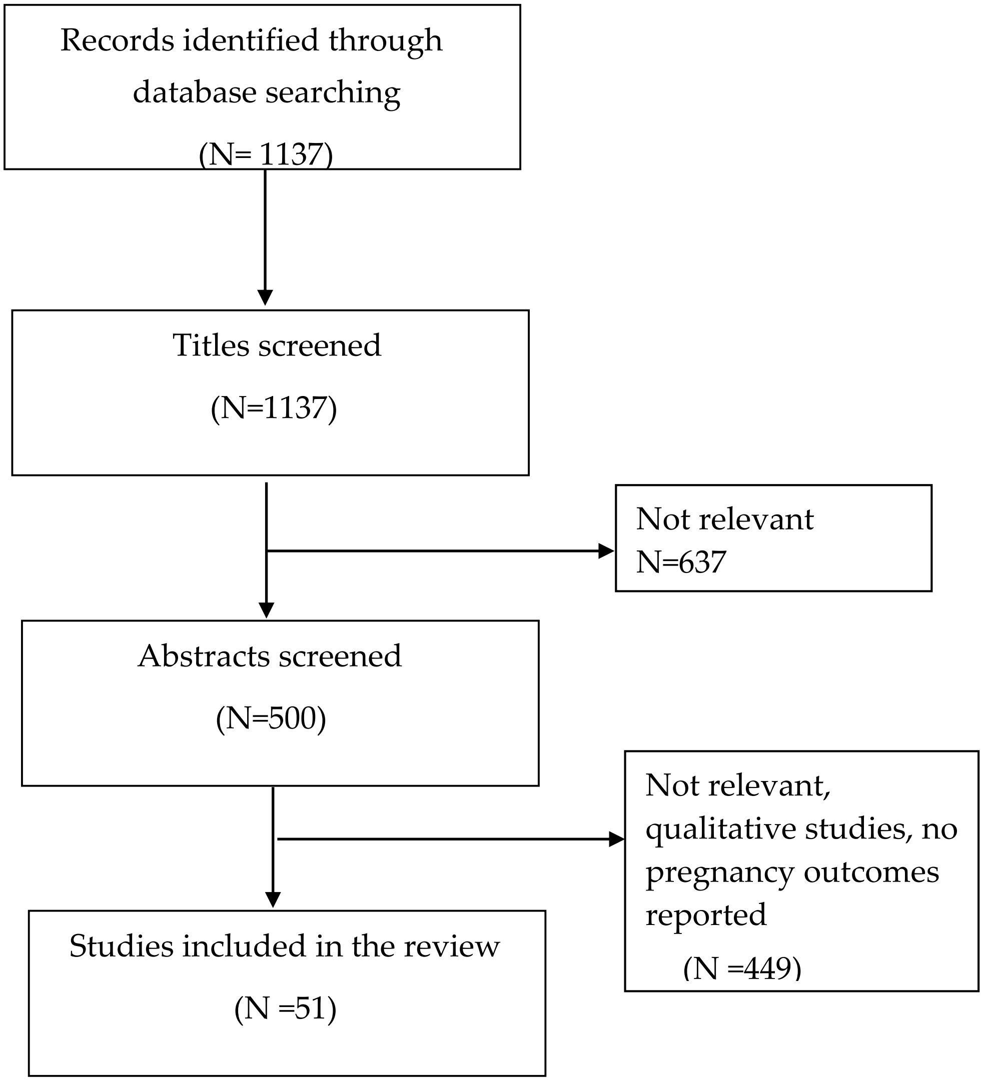

Figure S1. Flow chart for selection of studies reporting on IPV and pregnancy outcomes used in Table S1. The keywords "intimate partner violence" and "pregnancy" or "pregnant" were used. Studies reporting on IPV and pregnancy outcomes were included.

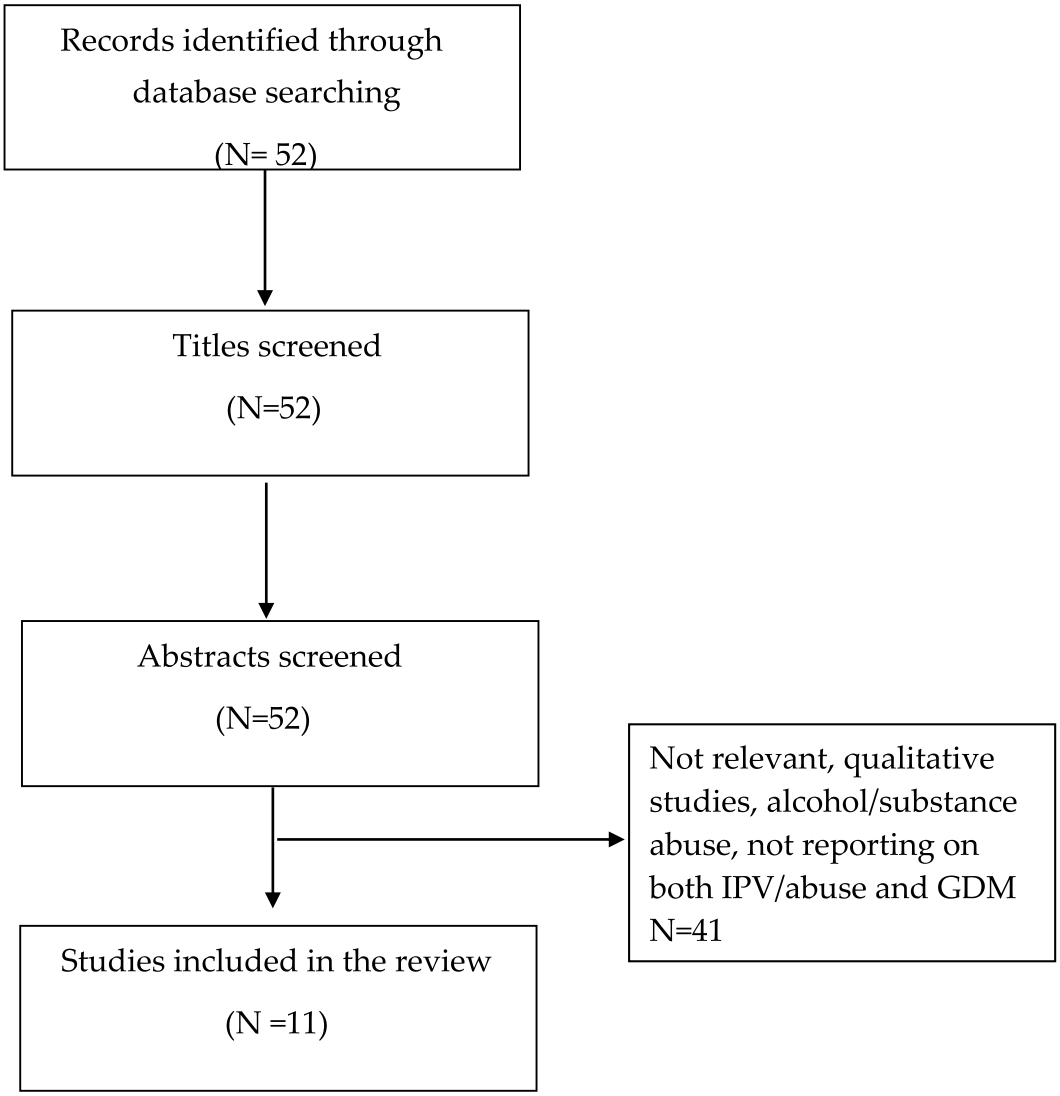

Figure S2. Flow chart for selection of studies reporting on IPV/abuse and GDM used in Table 2. The keywords "intimate partner violence" or "abuse" and "gestational diabetes" were used. Studies reporting on IPV or abuse and GDM were included.

References

Abdollahi, F., Abhari, F. R., Delavar, M. A., and Charati, J. Y. (2015). Physical violence against pregnant women by an intimate partner, and adverse pregnancy outcomes in Mazandaran Province, Iran. *J Family Community Med* 22, 13–18. doi:10.4103/2230-8229.149577.

Alhusen, J. L., Bullock, L., Sharps, P., Schminkey, D., Comstock, E., and Campbell, J. (2014). Intimate partner violence during pregnancy and adverse neonatal outcomes in low-income women. *J Womens Health (Larchmt)* 23, 920–926. doi:10.1089/jwh.2014.4862.

Belay, S., Astatkie, A., Emmelin, M., and Hinderaker, S. G. (2019). Intimate partner violence and maternal depression during pregnancy: A community-based cross-sectional study in Ethiopia. *PLoS One* 14, e0220003. doi:10.1371/journal.pone.0220003.

Berhanie, E., Gebregziabher, D., Berihu, H., Gerezgiher, A., and Kidane, G. (2019). Intimate partner violence during pregnancy and adverse birth outcomes: a case-control study. *Reprod Health* 16, 22. doi:10.1186/s12978-019-0670-4.

Coker, A. L., Sanderson, M., and Dong, B. (2004). Partner violence during pregnancy and risk of adverse pregnancy outcomes. *Paediatr Perinat Epidemiol* 18, 260–269. doi:10.1111/j.1365-3016.2004.00569.x.

Dahlen, H. G., Munoz, A. M., Schmied, V., and Thornton, C. (2018). The relationship between intimate partner violence reported at the first antenatal booking visit and obstetric and perinatal outcomes in an ethnically diverse group of Australian pregnant women: a population-based study over 10 years. *BMJ Open* 8, e019566. doi:10.1136/bmjopen-2017-019566.

Demelash, H., Nigatu, D., and Gashaw, K. (2015). A Case-Control Study on Intimate Partner Violence during Pregnancy and Low Birth Weight, Southeast Ethiopia. *Obstet Gynecol Int* 2015, 394875. doi:10.1155/2015/394875.

Donovan, B. M., Spracklen, C. N., Schweizer, M. L., Ryckman, K. K., and Saftlas, A. F. (2016). Intimate partner violence during pregnancy and the risk for adverse infant outcomes: a systematic review and meta-analysis. *BJOG* 123, 1289–1299. doi:10.1111/1471-0528.13928.

Fanslow, J., Silva, M., Whitehead, A., and Robinson, E. (2008). Pregnancy outcomes and intimate partner violence in New Zealand. *Aust N Z J Obstet Gynaecol* 48, 391–397. doi:10.1111/j.1479-828X.2008.00866.x.

Ferdos, J., Rahman, M. M., Jesmin, S. S., Rahman, M. A., and Sasagawa, T. (2018). Association between intimate partner violence during pregnancy and maternal pregnancy complications among recently delivered women in Bangladesh. *Aggress Behav* 44, 294–305. doi:10.1002/ab.21752.

Field, S., Onah, M., van Heyningen, T., and Honikman, S. (2018). Domestic and intimate partner violence among pregnant women in a low resource setting in South Africa: a facility-based, mixed methods study. *BMC Womens Health* 18, 119–119. doi:10.1186/s12905-018-0612-2.

Finnbogadóttir, H., Baird, K., and Thies-Lagergren, L. (2020). Birth outcomes in a Swedish population of women reporting a history of violence including domestic violence during pregnancy: a longitudinal cohort study. *BMC Pregnancy Childbirth* 20, 183. doi:10.1186/s12884-020-02864-5.

FitzPatrick, K. M., Brown, S., Hegarty, K., Mensah, F., and Gartland, D. (2020). Physical and Emotional Intimate Partner Violence and Women's Health in the First Year After Childbirth: An Australian Pregnancy Cohort Study. *J Interpers Violence*, 886260520934426. doi:10.1177/0886260520934426.

Gebrekristos, L. T., Groves, A. K., McNaughton Reyes, L., Maman, S., and Moodley, D. (2020). IPV victimization in pregnancy increases postpartum STI incidence among adolescent mothers in Durban, South Africa. *AIDS Care* 32, 193–197. doi:10.1080/09540121.2020.1742871.

Gebreslasie, K. Z., Weldemariam, S., Gebre, G., and Mehari, M.-A. (2020). Intimate partner violence during pregnancy and risk of still birth in hospitals of Tigray region Ethiopia. *Ital J Pediatr* 46, 107. doi:10.1186/s13052-020-00857-w.

Habtamu Belete, A., Alemayehu Assega, M., Alemu Abajobir, A., Abebe Belay, Y., and Kassahun Tariku, M. (2019). Prevalence of antenatal depression and associated factors among pregnant women in Aneded woreda, North West Ethiopia: a community based cross-sectional study. *BMC Res Notes* 12, 713. doi:10.1186/s13104-019-4717-y.

Han, A., and Stewart, D. E. (2014). Maternal and fetal outcomes of intimate partner violence associated with pregnancy in the Latin American and Caribbean region. *Int J Gynaecol Obstet* 124, 6–11. doi:10.1016/j.ijgo.2013.06.037.

Hassan, M., Kashanian, M., Hassan, M., Roohi, M., and Yousefi, H. (2014). Maternal outcomes of intimate partner violence during pregnancy: study in Iran. *Public Health* 128, 410–415. doi:10.1016/j.puhe.2013.11.007.

Hill, A., Pallitto, C., McCleary-Sills, J., and Garcia-Moreno, C. (2016). A systematic review and meta-analysis of intimate partner violence during pregnancy and selected birth outcomes. *Int J Gynaecol Obstet* 133, 269–276. doi:10.1016/j.ijgo.2015.10.023.

Hoang, T. N., Van, T. N., Gammeltoft, T., W Meyrowitsch, D., Nguyen Thi Thuy, H., and Rasch, V. (2016). Association between Intimate Partner Violence during Pregnancy and Adverse Pregnancy Outcomes in Vietnam: A Prospective Cohort Study. *PLoS One* 11, e0162844. doi:10.1371/journal.pone.0162844.

Islam, M. J., Broidy, L., Baird, K., and Mazerolle, P. (2017a). Exploring the associations between intimate partner violence victimization during pregnancy and delayed entry into prenatal care: Evidence from a population-based study in Bangladesh. *Midwifery* 47, 43–52. doi:10.1016/j.midw.2017.02.002.

Islam, M. J., Broidy, L., Baird, K., and Mazerolle, P. (2017b). Intimate partner violence around the time of pregnancy and postpartum depression: The experience of women of Bangladesh. *PLoS One* 12, e0176211. doi:10.1371/journal.pone.0176211.

Jamieson, B. (2020). Exposure to Interpersonal Violence During Pregnancy and Its Association With Women's Prenatal Care Utilization: A Meta-Analytic Review. *Trauma Violence Abuse* 21, 904–921. doi:10.1177/1524838018806511.

Janssen, P. A., Holt, V. L., Sugg, N. K., Emanuel, I., Critchlow, C. M., and Henderson, A. D. (2003). Intimate partner violence and adverse pregnancy outcomes: a population-based study. *Am J Obstet Gynecol* 188, 1341–1347. doi:10.1067/mob.2003.274.

Jaraba, S. M. R., and Garcés-Palacio, I. C. (2019). Association between violence during pregnancy and preterm birth and low birth weight in Colombia: Analysis of the demographic and health survey. *Health Care Women Int* 40, 1149–1169. doi:10.1080/07399332.2019.1566331.

Kaiyo-Utete, M., Dambi, J. M., Chingono, A., Mazhandu, F. S. M., Madziro-Ruwizhu, T. B., Henderson, C., et al. (2020). Antenatal depression: an examination of prevalence and its associated factors among pregnant women attending Harare polyclinics. *BMC Pregnancy Childbirth* 20, 197. doi:10.1186/s12884-020-02887-y.

Khan, S. F., Dongarwar, D., Aliyu, M. H., and Salihu, H. M. (2019). Multicountry analysis of pregnancy termination and intimate partner violence in Latin America using Demographic and Health Survey data. *Int J Gynaecol Obstet* 146, 296–301. doi:10.1002/ijgo.12876.

Komori, K., Komori, M., Eitoku, M., Joelle Muchanga, S. M., Ninomiya, H., Kobayashi, T., et al. (2019). Verbal abuse during pregnancy increases frequency of newborn hearing screening referral: The Japan Environment and Children's Study. *Child Abuse Negl* 90, 193–201. doi:10.1016/j.chiabu.2019.01.025.

Laelago, T., Belachew, T., and Tamrat, M. (2017). Effect of intimate partner violence on birth outcomes. *Afr Health Sci* 17, 681–689. doi:10.4314/ahs.v17i3.10.

Leone, J. M., Lane, S. D., Koumans, E. H., DeMott, K., Wojtowycz, M. A., Jensen, J., et al. (2010). Effects of intimate partner violence on pregnancy trauma and placental abruption. *J Womens Health (Larchmt)* 19, 1501–1509. doi:10.1089/jwh.2009.1716.

Ludermir, A. B., Lewis, G., Valongueiro, S. A., de Araújo, T. V. B., and Araya, R. (2010). Violence against women by their intimate partner during pregnancy and postnatal depression: a prospective cohort study. *Lancet* 376, 903–910. doi:10.1016/S0140-6736(10)60887-2.

Mahenge, B., Likindikoki, S., Stöckl, H., and Mbwambo, J. (2013). Intimate partner violence during pregnancy and associated mental health symptoms among pregnant women in Tanzania: a cross-sectional study. *BJOG* 120, 940–946. doi:10.1111/1471-0528.12185.

Martin-de-Las-Heras, S., Velasco, C., Caño, A., Luna-Del-Castillo, J. de D., and Khan, K. S. (2019a). Poor antenatal care attendance is associated with intimate partner violence: Multivariate analysis of a pregnancy cohort. *Eur J Obstet Gynecol Reprod Biol* 237, 204–208. doi:10.1016/j.ejogrb.2019.05.001.

Martin-de-Las-Heras, S., Velasco, C., Luna-Del-Castillo, J. D., and Khan, K. S. (2019b). Breastfeeding avoidance following psychological intimate partner violence during pregnancy: a cohort study and multivariate analysis. *BJOG* 126, 778–783. doi:10.1111/1471-0528.15592.

Martin-de-las-Heras, S., Velasco, C., Luna-del-Castillo, J. de D., and Khan, K. S. (2019). Maternal outcomes associated to psychological and physical intimate partner violence during pregnancy: A cohort study and multivariate analysis. *PLOS ONE* 14, e0218255. doi:10.1371/journal.pone.0218255.

Miura, A., and Fujiwara, T. (2017). Intimate Partner Violence during Pregnancy and Postpartum Depression in Japan: A Cross-sectional Study. *Front Public Health* 5, 81. doi:10.3389/fpubh.2017.00081.

Ogunwale, A. N., Anderson, M. L., and Sangi-Haghpeykar, H. (2017). Intimate Partner Violence During Pregnancy and Maternal and Neonatal Health Among Hispanic Women. *J Reprod Med* 62, 65–71.

Pastor-Moreno, G., Ruiz-Pérez, I., Henares-Montiel, J., Escribà-Agüir, V., Higueras-Callejón, C., and Ricci-Cabello, I. (2020a). Intimate partner violence and perinatal health: a systematic review. *BJOG* 127, 537–547. doi:10.1111/1471-0528.16084.

Pastor-Moreno, G., Ruiz-Pérez, I., Henares-Montiel, J., and Petrova, D. (2020b). Intimate partner violence during pregnancy and risk of fetal and neonatal death: a meta-analysis with socioeconomic context indicators. *Am J Obstet Gynecol* 222, 123-133.e5. doi:10.1016/j.ajog.2019.07.045.

Rogathi, J. J., Manongi, R., Mushi, D., Rasch, V., Sigalla, G. N., Gammeltoft, T., et al. (2017). Postpartum depression among women who have experienced intimate partner violence: A prospective cohort study at Moshi, Tanzania. *J Affect Disord* 218, 238–245. doi:10.1016/j.jad.2017.04.063.

Rurangirwa, A. A., Mogren, I., Ntaganira, J., Govender, K., and Krantz, G. (2018). Intimate partner violence during pregnancy in relation to non-psychotic mental health disorders in Rwanda: a cross-sectional population-based study. *BMJ Open* 8, e021807. doi:10.1136/bmjopen-2018-021807.

Sanchez, S. E., Alva, A. V., Diez Chang, G., Qiu, C., Yanez, D., Gelaye, B., et al. (2013). Risk of spontaneous preterm birth in relation to maternal exposure to intimate partner violence during pregnancy in Peru. *Matern Child Health J* 17, 485–492. doi:10.1007/s10995-012-1012-0.

Shamu, S., Munjanja, S., Zarowsky, C., Shamu, P., Temmerman, M., and Abrahams, N. (2018). Intimate partner violence, forced first sex and adverse pregnancy outcomes in a sample of Zimbabwean women accessing maternal and child health care. *BMC Public Health* 18, 595. doi:10.1186/s12889-018-5464-z.

Sigalla, G. N., Mushi, D., Meyrowitsch, D. W., Manongi, R., Rogathi, J. J., Gammeltoft, T., et al. (2017). Intimate partner violence during pregnancy and its association with preterm birth and low birth weight in Tanzania: A prospective cohort study. *PLoS One* 12, e0172540. doi:10.1371/journal.pone.0172540.

Singh, J. K., Evans-Lacko, S., Acharya, D., Kadel, R., and Gautam, S. (2018). Intimate partner violence during pregnancy and use of antenatal care among rural women in southern Terai of Nepal. *Women Birth* 31, 96–102. doi:10.1016/j.wombi.2017.07.009.

Stöckl, H., Hertlein, L., Himsl, I., Delius, M., Hasbargen, U., Friese, K., et al. (2012). Intimate partner violence and its association with pregnancy loss and pregnancy planning. *Acta Obstet Gynecol Scand* 91, 128–133. doi:10.1111/j.1600-0412.2011.01264.x.

Suzuki, S., Yamada, F., and Eto, M. (2018). Influence of intimate partner violence on mental status in Japanese women during the first trimester of pregnancy. *J Matern Fetal Neonatal Med* 31, 1524–1526. doi:10.1080/14767058.2017.1317739.

Tho Nhi, T., Hanh, N. T. T., Hinh, N. D., Toan, N. V., Gammeltoft, T., Rasch, V., et al. (2019). Intimate Partner Violence among Pregnant Women and Postpartum Depression in Vietnam: A Longitudinal Study. *Biomed Res Int* 2019, 4717485. doi:10.1155/2019/4717485.

Thomas, J. L., Lewis, J. B., Martinez, I., Cunningham, S. D., Siddique, M., Tobin, J. N., et al. (2019). Associations between intimate partner violence profiles and mental health among low-income, urban pregnant adolescents. *BMC Pregnancy Childbirth* 19, 120. doi:10.1186/s12884-019-2256-0.

Tiruye, T. Y., Chojenta, C., Harris, M. L., Holliday, E., and Loxton, D. (2020). Intimate partner violence against women and its association with pregnancy loss in Ethiopia: evidence from a national survey. *BMC Womens Health* 20, 192. doi:10.1186/s12905-020-01028-z.

Yu, H., Jiang, X., Bao, W., Xu, G., Yang, R., and Shen, M. (2018). Association of intimate partner violence during pregnancy, prenatal depression, and adverse birth outcomes in Wuhan, China. *BMC Pregnancy Childbirth* 18, 469–469. doi:10.1186/s12884-018-2113-6.
